# Commentary: Ultrasound-Based Scoring System for Indication of Pyeloplasty in Patients With UPJO-Like Hydronephrosis

**DOI:** 10.3389/fped.2020.594527

**Published:** 2020-12-03

**Authors:** Abdurrahman Onen

**Affiliations:** Section of Pediatric Urology, Department of Pediatric Surgery, Faculty of Medicine, Dicle University, Diyarbakir, Turkey

**Keywords:** Hydronephrosis, ultrasound scoring, Onen grading, pyeloplasty, UPJ-type obstruction

## Introduction

A recent study has suggested a pyeloplasty prediction score (PPS) using three ultrasound parameters to determine who need surgery and who do not in infants (<3 years old) with ureteropelvic junction obstruction (UPJO)-like hydronephrosis (1). They recommend a combination of SFU grade (A), transverse AP diameter (B) and the absolute percentage difference of ipsilateral and contralateral renal lengths at baseline (C) to predict a criteria for surgical need. This study suggests that any infant UPJO-like hydronephrosis with a PPS of 8 or higher are 8 times more likely to undergo pyeloplasty (1). Unfortunately, none of these parameters is ideal to use due to many disadvantages and/or limitations (2). When we put problematic parameters together it is unlikely to get a correct beneficial result from them.

## SFU Grading System (A)

All grades of SFU are very variable between operators and clinicians (2–7). *SFU-3* represents only caliceal dilation which does not cause renal damage unless increase in hydronephrosis or development of any symptom (2, 3, 8, 9). Therefore, SFU-3 by itself, should not be an indication for pyeloplasty. *SFU-4* represents minimal thinning of medullary parenchyma (ex. 6 mm) and severe thinning of cortical parenchyma (ex. 2 mm) and cyst-like hydronephrotic kidneys at the same grade (2, 3, 8, 9). This wide definition of SFU-4 is failure to demonstrate accurately the severity of hydronephrosis and thus a significant misleading for prompt treatment (2, 3, 8, 10).

## Anterior-Posterior (AP) Diameter of Renal Pelvis (B)

It is a very dynamic parameter that change significantly depending on operator, hydration, bladder filling, position (supin or prone), and respiration (2–4, 11). More importantly its measurement is very variable and misleading due to different renal pelvic configurations (2–4). Hydronephrosis may be moderate even if the AP diameter is high in infants with extrarenal pelvic configuration. On the other hand, hydronephrosis may be very severe with significant parenchymal thinning even if the AP diameter is low in infants with intrarenal pelvic configuration. In the literature, there is no study determining intra- and inter-observer reproducibility of the measurement of AP diameter.

## The Absolute Percentage Difference of Ipsilateral and Contralateral Renal Lengths (C)

- The laterality may significantly change the results of absolute percentage.
  *Example:* Normal kidney longitudinal length for an infant who is 11 months of age:
  ° Normal right kidney longitudinal length; 64.24 ± 2.64 mm. It means that right kidney may be 61.60 mm (4).
  ° Normal left kidney longitudinal length; 66.36 ± 2.41 mm. It means that left kidney may be 68.77 mm (4).
  If this infant has right UPJO-like hydronephrosis; C = 61.60–68.77 = −7.17! If this infant has left UPJO-like hydronephrosis; C = 68.77–61.60 = 7.17!
- Any degree of contralateral or bilateral hydronephrosis, ipsilateral atrophy, or contralateral hypertrophy will significantly change the absolute percentage (C).
  ° This percentage would be low when there is a contralateral compensatory growth which will miss the severity of hydronephrosis.
  ° Similarly, it would be low when there is an atrophy in ipsilateral kidney which, again, will miss the severity of hydronephrosis.
  ° In addition, how would it be an objective criteria in bilateral cases?
  Any of these parameters can change the percentage (C) from 5 to 20% which means that it may get a score from 0 to 4!

## Pyeloplasty Prediction Score (PPS)

Example-1

- A: SFU-4 (minimal medullary thinning with normal cortex)
- B: AP = 20 mm (extrarenal pelvic configuration)
- C: 17% (without ipsilateral atrophy or contralateral hypertrophy)

PPS = A + B + C = 4 + 4 + 3 = 11.


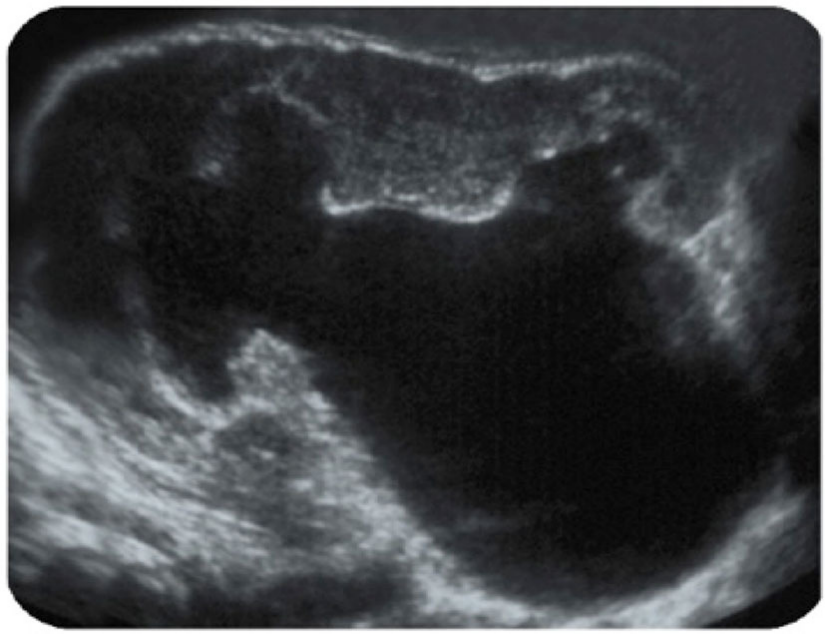


Parenchyma: 5.4 mm, AP diameter: 36 mm.

According to PPS, this patient clearly should undergo pyeloplasty. However, such a patient does not need surgery if there is no significant contralateral compensatory growth or ipsilateral atrophy or significant functional decrease.

Example-2

- A: SFU-4 (significant cortical thinning with/without hyperechogenecity)
- B: AP = 15 (intrarenal pelvic configuration)
- C: <5% (in the presence of ipsilateral atrophy and contralateral hypertrophy)

PPS = A + B + C = 4 + 2 + 0 = 6.


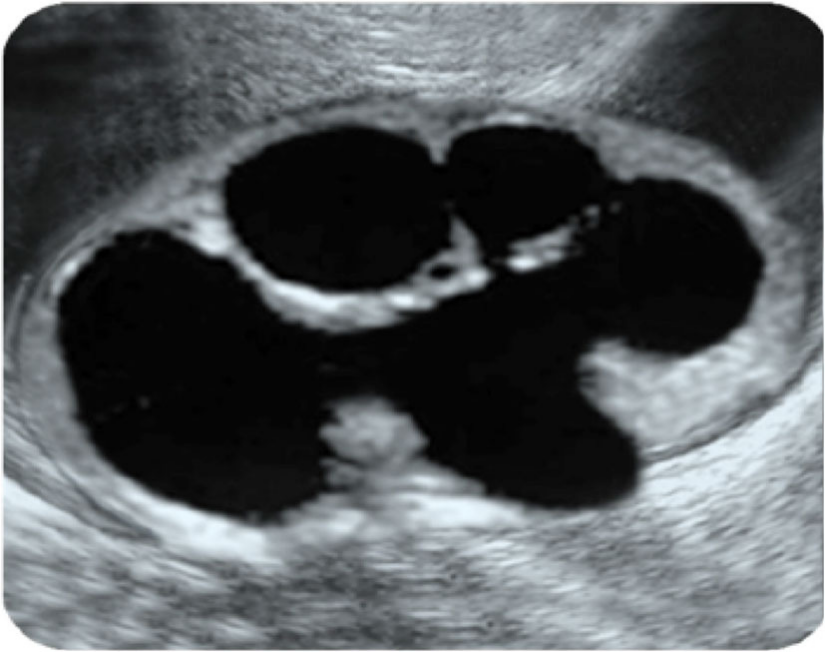


Parenchyma: 2.3 mm, AP diameter: 14 mm.

According to PPS, this patient should be followed conservatively. However, such a patient definitely need surgery. Otherwise irreversible renal damage will develop in this patient if pyeloplasty delayed for a few month.

## Discussion

The laterality (normal right and left long length is different), contralateral or bilateral hydronephrosis, ipsilateral atrophy or contralateral hypertrophy significantly change the results of pyeloplasty prediction score (A+B+C) (2). The absolute percentage (C) would be low when there is a contralateral compensatory growth or an atrophy in ipsilateral kidney which will miss the severity of hydronephrosis. In addition, it is not an objective criteria in bilateral cases. Any of these parameters can change the percentage (C) from 5 to 20% which means the score may change from 0 to 4. We should use objective and reproducible criteria that does not affect from many parameters and applicable for all patients.

Neither AP diameter nor SFU or the percentage of renal length are gold standard to determine the severity of hydronephrosis. Due to the fact that all parameters of PPS are affected by many factors, none of the PPS criteria is suitable or sufficient for standardizing UPJO-like hydronephrosis (2). They do not determine the exact severity of UPJO-like hydronephrosis and do not correctly reflect renal injury in UPJO because they do not take the quality of renal parenchyma into account. They, therefore, may cause permanent renal damage due to a delay in surgical decision in some infants while may cause an unnecessary surgery in others.

The anatomy and physiology of the 4 suborgans of the kidney (renal pelvis, calices, medulla, and cortex) are completely different from each other and each produces different risk of renal damage. Therefore, each part of kidney behave differently as a response to hydronephrosis.

The quality (*thickness* and *appearance)* of renal parenchyma is the most important and objective parameter to determine kidney exposure, renal function and thus the severity of hydronephrosis. Renal c*ortical thickness* is the most important functional part of kidney. It is an objective parameter because, opposite to pelvicaliceal system, it is not affected by hydration, bladder filling, position, and respiration. The measurement points are not controversial and is not operator dependant (2–4, 12). It does not have intra or inter observer variation (2, 4, 13). *Hyperechogene* parenchyma, *cystic cortical degeneration* and *loss of corticomedullary differentiation* on ultrasound are findings suggesting significant renal damage which are compatible with decrease in renal function on scintigraphy (2, 14).

Comparing the PPS criteria, Onen hydronephrosis grading system has evidence-based objective parameters to define the severity of UPJO-like hydronephrosis promptly (10). Onen grading system shows a significant relationship with renal histopathologic grade and thus can be an indicator for renal injury in UPJO-like hydronephrosis (10). It is a reliable, easily reproducible and play a significant role in the diagnosis of obstruction in children (2, 6). It does suggest who need surgery and who can safely be followed non-operatively (2).

## Author Contributions

The author confirms being the sole contributor of this work and has approved it for publication.

## Conflict of Interest

The author declares that the research was conducted in the absence of any commercial or financial relationships that could be construed as a potential conflict of interest.
